# Body-First Subtype of Parkinson’s Disease with Probable REM-Sleep Behavior Disorder Is Associated with Non-Motor Dominant Phenotype

**DOI:** 10.3233/JPD-223511

**Published:** 2022-12-16

**Authors:** Lukas Pavelka, Armin Rauschenberger, Zied Landoulsi, Sinthuja Pachchek, Taina Marques, Clarissa P.C. Gomes, Enrico Glaab, Patrick May, Rejko Krüger, on behalf of the NCER-PD Consortium

**Affiliations:** a Parkinson’s Research Clinic, Centre Hospitalier de Luxembourg (CHL), Luxembourg, Luxembourg; b Transversal Translational Medicine, Luxembourg Institute of Health (LIH), Strassen, Luxembourg; c Biomedical Data Science Group, Luxembourg Centre for Systems Biomedicine (LCSB), University of Luxembourg, Esch-sur-Alzette, Luxembourg; d Bioinformatics Core, Luxembourg Centre for Systems Biomedicine (LCSB), Esch-sur-Alzette, Luxembourg; e Translational Neuroscience, Luxembourg Centre for Systems Biomedicine (LCSB), University of Luxembourg, Esch-sur-Alzette, Luxembourg

**Keywords:** Idiopathic Parkinson’s disease, probable REM-Sleep behavior disorder, RBDSQ, non-motor symptoms, APOE, stratification

## Abstract

**Background:**

The hypothesis of *body-first* vs. *brain-first* subtype of PD has been proposed with REM-Sleep behavior disorder (RBD) defining the former. The body-first PD presumes an involvement of the brainstem in the pathogenic process with higher burden of autonomic dysfunction.

**Objective:**

To identify distinctive clinical subtypes of idiopathic Parkinson’s disease (iPD) in line with the formerly proposed concept of *body-first* vs. *brain-first* subtypes in PD, we analyzed the presence of probable RBD (pRBD), sex, and the *APOE* *ɛ*4 carrier status as potential sub-group stratifiers.

**Methods:**

A total of 400 iPD patients were included in the cross-sectional analysis from the baseline dataset with a completed RBD Screening Questionnaire (RBDSQ) for classifying as pRBD by using the cut-off RBDSQ≥6. Multiple regression models were applied to explore (i) the effect of pRBD on clinical outcomes adjusted for disease duration and age, (ii) the effect of sex on pRBD, and (iii) the association of *APOE* *ɛ*4 and pRBD.

**Results:**

iPD-pRBD was significantly associated with autonomic dysfunction (SCOPA-AUT), level of depressive symptoms (BDI-I), MDS-UPDRS I, hallucinations, and constipation, whereas significantly negatively associated with quality of life (PDQ-39) and sleep (PDSS). No significant association between sex and pRBD or *APOE* ɛ4 and pRBD in iPD was found nor did we determine a significant effect of *APOE* ɛ4 on the PD phenotype.

**Conclusion:**

We identified an RBD-specific PD endophenotype, characterized by predominant autonomic dysfunction, hallucinations, and depression, corroborating the concept of a distinctive *body-first* subtype of PD. We did not observe a significant association between *APOE* ɛ4 and pRBD suggesting both factors having an independent effect on cognitive decline in iPD.

## INTRODUCTION

The phenotypic heterogeneity of Parkinson’s disease (PD) has been a challenge for both clinicians and researchers for decades. Several efforts were made to identify an underlying pattern explaining this heterogeneity by subtyping PD patients. They can be grouped into two distinct methods. The first approach uses a single clinical or genetic metric determining the clinical phenotype, such as age at onset, sex, motor phenotype, or being a carrier of the PD-causing rare genetic mutations. The second approach has been using hypothesis-free data-driven models identifying phenotypic clusters in PD based on clinical symptoms, but this approach failed reproducibility checks, possibly due to a limited methodological overlap between the studies and a wide variety of clinical metrics entering the models [1]. Interestingly, both approaches systematically reported REM-sleep behavior disorder (RBD) as a relevant clinical variable. Not only is RBD currently known as the most robust prodromal marker of future pheno-conversion to the alpha-synucleinopathies (i.e., PD, dementia with Lewy bodies or multiple system atrophy) [2], but it was suggested that RBD is associated with more rapid progression of motor symptoms, a higher burden of non-motor symptoms and lower quality of life [3–5].

RBD received increasing attention in the last years, with several cross-sectional and longitudinal studies investigating the association between RBD and the clinical phenotype of PD. On the one hand, we observe an overall consensus regarding a non-motor dominant profile of PD with higher autonomic dysfunction and more rapid cognitive decline. On the other hand, prior studies have reported contradictory findings on the effect of comorbid RBD on motor progression in PD [5–8]. Moreover, genetic risk factors and PD-causing rare mutations with a substantial effect on the clinical phenotype were rarely systematically addressed in the context of concomitant RBD and PD and their effect on the severity of the clinical phenotype. Recently, the *APOE* epsilon4 (*APOE* ɛ4) genotype has been linked to faster cognitive decline and motor progression in PD [9], although studies on the role of *APOE* ɛ4 and clinical progression of PD remain controversial [10, 11]. Whether an additive or multiplicative potentiation effect of RBD and *APOE* ɛ4 on cognitive decline in PD exists has not been adequately addressed so far. Currently, no association of the *APOE* ɛ4 carriers status with idiopathic RBD has been observed [12, 13], but a potential role of the *APOE* ɛ4 genotype as a modifier of the clinical phenotype of PD with RBD has not yet been explored.

RBD has been suggested to represent a key element in distinguishing body-first from brain-first subtype of PD, a concept recently proposed to explain the phenotypic differences and variability of dynamics in PD and supported by several clinical and imaging studies [14, 15]. It has been proposed that the body-first subtype of PD starts in the peripheral nervous system with spreading of neurodegeneration via brainstem thus associated with RBD, higher burden of autonomic dysfunction and higher rate of cognitive decline [16].

In order to test the hypothesis of body-first subtype of PD with comorbid pRBD, we used a large baseline visit dataset from the Luxembourg Parkinson’s Study, a monocentric longitudinal observational study with a previously described recruitment design [17]. In our study, we primarily aimed to determine the effect of pRBD on clinical outcomes in idiopathic PD (iPD) by excluding known PD-linked rare mutations or genetic risk variant carriers. Next, we investigated potential confounding effects of sex and the *APOE* ɛ4 carrier status as potential stratifiers of iPD.

## MATERIALS AND METHODS

### Study population

**Fig. 1:**
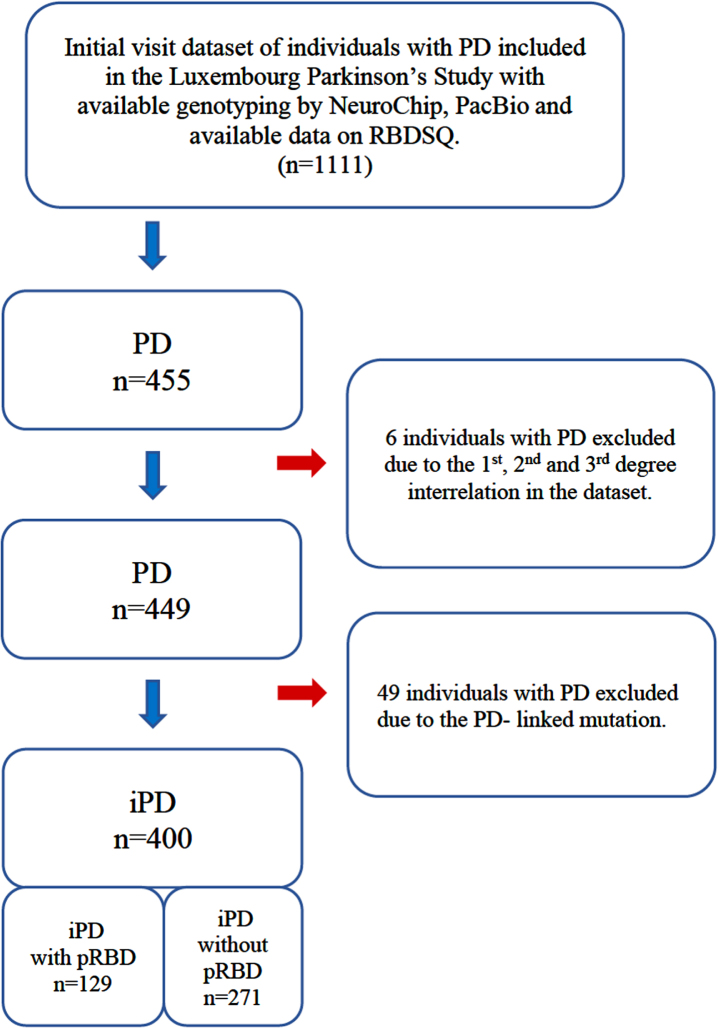
Description of the study design and study dataset. PD, individuals with Parkinson’s disease; iPD, idiopathic Parkinson’s disease; pRBD, probable REM-sleep behavior disorder; RBDSQ, REM sleep behavior disorder screening questionnaire.

The data used in this study were acquired from participants recruited in the frame of the nationwide monocentric observational longitudinal Luxembourg Parkinson’s Study [17]. The diagnosis of PD relied on the UK Parkinson’s Disease Society Brain Bank (UKPDSBB) diagnostic criteria [18]. All participants were genotyped for disease-causing mutations and PD-associated risk variants using both NeuroChip^®^ and PacBio sequencing. Available data on RBDSQ were analyzed after excluding six PD patients for 1st, 2nd, and 3rd degree relationships and after excluding 49 PD patients carrying PD-associated mutations. The overall study design, inclusion, and exclusion workflow are illustrated in Fig. 1. Though the diagnostic gold standard of RBD remains polysomnography (PSG) [19], the accessibility of the sleep laboratory and performing PSG on a large scale is problematic due to the sleep laboratory capacities and costs. We therefore applied a classification of probable RBD (pRBD) by REM-sleep behavior disorder screening questionnaire (RBDSQ) as used in several previous studies [20–24]. The group assignment of pRBD in iPD individuals uses the criterion RBDSQ≥6 to optimize the specificity and sensitivity for pRBD in line with the Oxford Discovery Study [24].

All participants taking part in the Luxembourg Parkinson’s Study agreed and signed a written informed consent. The study has been approved by the National Research Ethics Committee (CNER Ref: 201407/13).

### Clinical assessment and data

The design and recruitment of the Luxembourg Parkinson’s Study were previously published in detail [17]. Sociodemographic characteristics and clinical outcomes validated for PD were chosen from the basic clinical assessment battery and are listed in Tables 1 and 2. All patients have been evaluated in medication ON state and, where applicable, in deep brain stimulation ON state. The clinical symptoms as scales are defined in detail in the Supplementary Material.

**Table 1:** Descriptive and comparative statistics of demographic data and frequency of *APOE* ɛ4 genotype in PD individuals with (right) and without (left) probable REM-sleep behavior disorder (pRBD). For intergroup comparisons, *p*-values are shown from Mann-Whitney *U* test for numerical variables and Fisher’s exact test for binary variables. Binary variables are annotated by asterisk. Results are shown as mean and standard deviation (SD) for numerical variables, number of zeros (’NO’) and ones (’YES’) for binary variables and percentage of YES, and number of missing values (NA). Single and double ticks indicate significance at the 5% level, and the Bonferroni-adjusted 5% level. Age at onset was calculated based on the year of the PD diagnosis. PD, Parkinson’s disease

	PD non-pRBD (*n* = 271)			PD pRBD (*n* = 129)		
	Mean or	SD or	NA	Mean or	SD or	NA	*p*
	YES in %	NO/YES		YES in %	NO/YES
Disease duration since diagnosis (y)	4.20	4.55	0	7.86	6.36	0	8.2e-11^′′^
Age at assessment (y)	66.19	11.29	0	68.31	9.85	0	1.2e-01
Age at onset (y)	62.01	11.64	0	60.48	11.98	0	2.5e-01
Sex (male)*	65%	96/175	0	74%	34/95	0	8.6e-02
*APOE* (ɛ2/ ɛ4; ɛ3/ ɛ4;ɛ4/ɛ4)*	21%	213/58	0	26%	95/34	0	3.1e-01
Years of education	13.29	4.12	0	12.99	3.90	0	6.7e-01
Total languages spoken	2.86	1.06	0	2.89	1.04	0	8.0e-01

**Table 2:** Descriptive and comparative statistics of clinical outcomes for iPD group with and without probable REM-sleep behavior disorder (pRBD). Results are shown as mean and standard deviation (SD) for numerical variables, number of zeros (’NO’) and ones (’YES’) for binary variables and percentage of YES, and number of missing values (NA). For intergroup comparisons, *p*-values are shown from Mann-Whitney *U* test for numerical variables and Fisher’s exact test for binary variables. Binary variables are annotated by asterisk. Single and double ticks indicate significance at the 5% level, and the Bonferroni-adjusted 5% level. All clinical outcomes are defined and described in the Supplementary Material

	PD non-pRBD (*n* = 271)			PD pRBD (*n* = 129)		
	Mean or	SD or	NA	Mean or	SD or	NA	*p*
	YES in %	NO/YES		YES in %	NO/YES
H&Y	2.12	0.78	2	2.37	0.75	0	1.2e-04^′′^
MDS-UPDRS III	32.00	16.11	5	38.02	16.76	2	4.5e-04^′′^
MDS-UPDRS II	9.79	7.45	3	14.50	8.64	3	1.0e-07^′′^
LEDD (g/day)	0.45	0.38	0	0.68	0.41	0	2.8e-08^′′^
Gait disorder*	48%	141/130	0	71%	37/92	0	1.0e-05^′′^
Repetitive falls*	11%	240/31	0	29%	91/38	0	1.7e-05^′′^
MDS-UPDRS IV	1.37	3.01	2	2.75	3.98	3	5.2e-05^′′^
Dyskinesia/day (hours)	0.47	2.29	0	1.21	3.57	1	9.3e-05^′′^
OFF time/day (hours)	0.40	1.41	0	0.72	1.38	2	3.2e-04^′′^
Dystonia/day (hours)	0.027	0.15	1	0.088	0.31	1	7.3e-03^′^
Dyskinesia*	9%	246/25	0	20%	103/26	0	3.5e-03^′^
Motor fluctuations*	11%	241/30	0	27%	94/35	0	8.1e-05^′′^
Freezing of gait*	16%	227/44	0	34%	85/44	0	9.4e-05^′′^
MoCA	24.85	3.93	5	24.02	4.45	2	6.9e-02
Sniffin’ stick test	8.52	3.34	7	7.50	3.27	3	1.0e-02^′^
PDQ-39	33.65	23.88	12	52.23	27.05	6	7.2e-11^′′^
SCOPA-AUT	12.59	6.97	2	19.59	8.11	0	6.7e-15^′′^
MDS-UPDRS I	8.54	5.78	6	13.62	7.36	4	5.1e-12^′′^
BDI-I	8.79	6.65	7	12.62	7.33	3	6.2e-08^′′^
Starkstein Apathy Scale	13.46	5.31	4	14.67	6.24	3	1.2e-01
PDSS	111.40	21.55	4	92.64	23.05	3	2.3e-13^′′^
Probable RBD*	0%	271/0	0	100%	0/129	0	1.4e-108^′′^
Excessive daily sleepiness*	23%	208/63	0	41%	76/53	0	3.8e-04^′′^
Insomnia*	24%	205/66	0	21%	102/27	0	5.3e-01
Hallucinations*	9%	247/24	0	29%	91/38	0	4.8e-07^′′^
Impulse Control Disorder*	6%	255/16	0	16%	108/21	0	1.4e-03^′^
Orthostatic hypotension*	23%	210/61	0	36%	82/47	0	3.9e-03^′^
Dysphagia*	20%	218/53	0	33%	87/42	0	5.6e-03^′^
Constipation*	31%	187/84	0	63%	48/81	0	2.8e-09^′′^
Urinary Incontinence*	27%	197/74	0	39%	79/50	0	2.8e-02^′^

### Missing data statement

The absolute number of missing data per variable is described in Tables 1 and 2. Given the low proportions of missing values in the dataset, we used a pairwise deletion for all statistical models.

### Genotyping and quality-control analyses

The methods for genotyping in our dataset have been described previously [25]. PD causing rare variants were defined by the ClinVar classification as “pathogenic/likely pathogenic”. All PD-causing variants (listed in the Supplementary Material) identified by any method were Sanger validated, and all samples with a validated PD-causing variant were excluded from further analysis with a list of excluded variants described in the Supplementary Material.

### APOE genotyping

*APOE* genotypes were called for all individuals from two SNPs investigated by NeuroChip array (rs429358, rs7412) that distinguish the ɛ2, ɛ3, and ɛ4 alleles classifying the respective *APOE* carriers. The NeuroChip provides high accuracy of 98.1% for genotyping of *APOE* ɛ4 [26], and the approach was aligned with other large studies [27].

### Statistical analysis

Mann-Whitney’s *U* test was used for numerical variables and Fisher’s exact test for binary variables in intergroup comparison analyses (iPD pRBD vs. iPD non-pRBD; male sex iPD vs. female sex iPD). Multiple linear and logistic regression models were applied to investigate the effect of pRBD on clinical outcomes in iPD, adjusted for age at assessment (AAA) and disease duration. To investigate the potential effect of the *APOE* genotype on clinical outcomes, we pooled the heterozygotes (ɛ2/ɛ4; ɛ3/ɛ4) and homozygotes (ɛ4/ɛ4), allowing us to quantify a potential association between *APOE* ɛ4 genotype and pRBD in iPD. Furthermore, we applied regression of clinical symptoms in PD on *APOE* ɛ4, AAA and disease duration. For all analyses, we assessed significance at the 5% level and the Bonferroni-adjusted 5% level.

## RESULTS

### Frequency of pRBD and effect of pRBD on clinical outcomes in iPD

According to the RBDSQ classification of pRBD, we observed a relative pRBD frequency of 32.3% in the iPD group (129 iPD pRBD out of 400). The demographic characteristics of iPD pRBD (*n* = 129) and iPD non-pRBD patients (*n* = 271) are shown in Tables 1 and 2. We investigated the effect of pRBD on the clinical outcomes adjusted for AAA and disease duration.

**Fig. 2:**
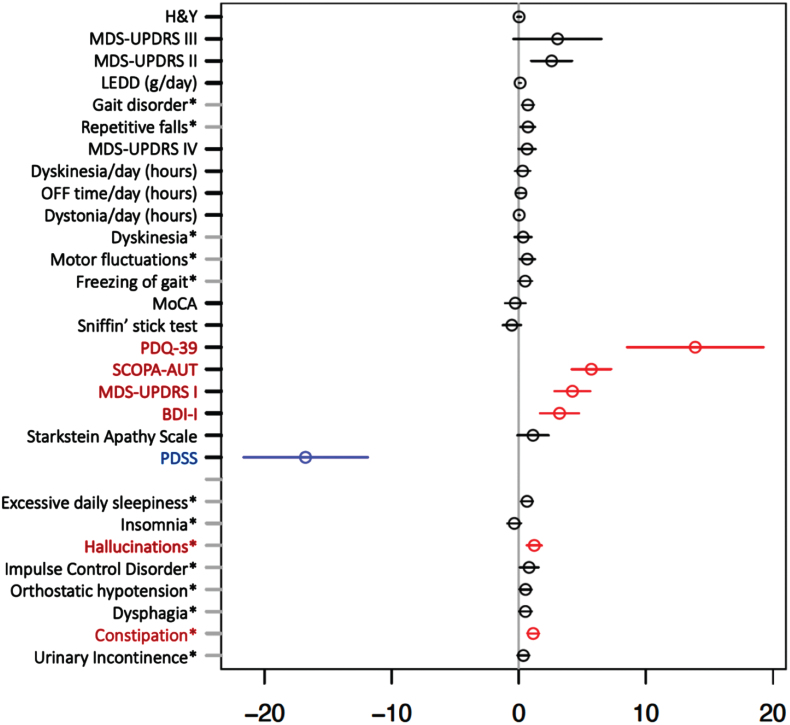
Multiple regression model for investigating effect of probable REM-Sleep behavior disorder on clinical outcomes in idiopathic Parkinson’s disease adjusted for age at assessment and disease duration. Forrest plot with estimated coefficients and corresponding confidence intervals (±1.96 x standard error) for pRBD, from linear/logistic regression of numerical/binary outcome on disease duration, age at assessment (AAA) and pRBD (binary outcomes are annotated by asterisk). The color blue indicates significant negative effects of pRBD on the clinical outcome, and the color red indicates significant positive effects at the Bonferroni-adjusted 5% level. Clinical symptoms and scales are described in the Supplementary Material.

As key results, we observed a significant positive association between iPD pRBD (as opposed to iPD non-pRBD) and burden of non-motor symptoms, i.e., autonomic dysfunction (SCOPA-AUT) and frequency of constipation; MDS-UPDRS I, burden of depression symptoms assessed by BDI-I, frequency of hallucinations and PDQ-39, showing lower quality of life in iPD pRBD, as demonstrated in Fig. 2. Furthermore, a significant negative association was determined between iPD pRBD and the Parkinson’s Disease Sleep Scale (PDSS), indicating lower quality of sleep in the group of iPD pRBD vs. iPD non-pRBD. Other considered clinical outcomes showed no significant associations after multiple testing correction.

### APOE genotype and iPD pRBD

**Fig. 3:**
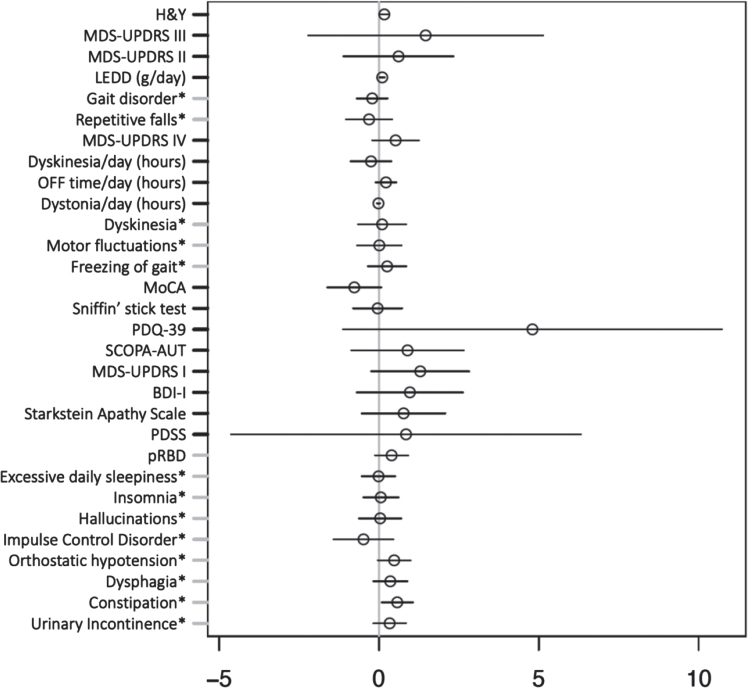
Multiple regression model investigating effect of *APOE* ɛ4 carrier status on clinical outcomes in idiopathic Parkinson’s disease adjusted for age at assessment and disease duration. Forrest plot with estimated coefficients and corresponding confidence intervals (±1.96 x standard error) for *APOE* ɛ4 genotype, from linear/logistic regression of numerical/binary outcome on disease duration, age at assessment (AAA), and *APOE* (binary outcomes are annotated by asterisk). The color blue indicates significant negative effects of *APOE* ɛ4 genotype on the clinical outcome, and the color red indicates significant positive effects at the Bonferroni-adjusted 5% level. Clinical symptoms and scales are described in the Supplementary Material.

We found no significant association between pooled heterozygote and homozygote *APOE* ɛ4 carriers and iPD with pRBD. Additionally, no significant association was observed between *APOE* ɛ4 and the clinical outcomes of iPD with pRBD vs. iPD non-pRBD adjusted for AAA and disease duration, as shown in Fig. 3.

### Effect of sex on frequency of pRBD and other clinical outcomes in iPD

**Fig. 4:**
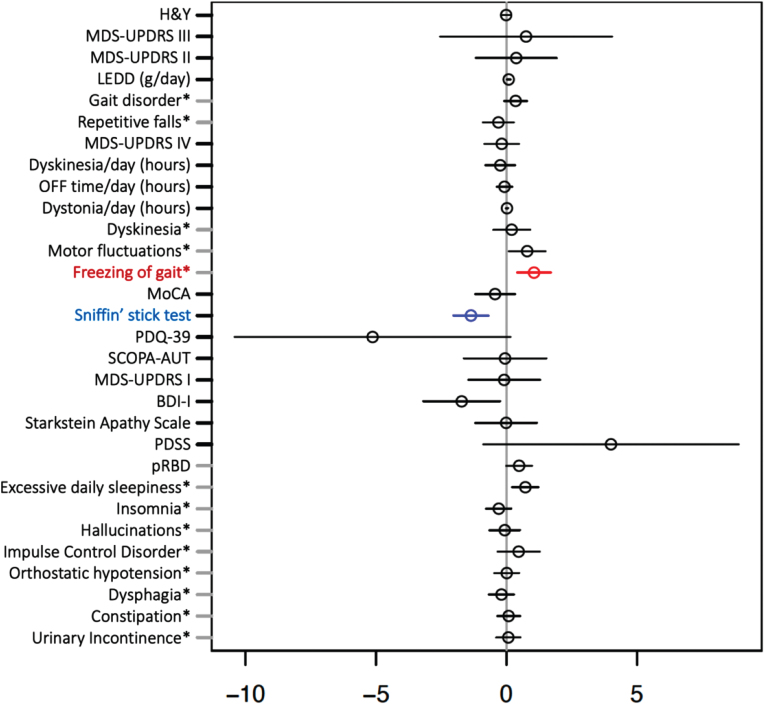
Multiple regression model investigating effect of sex on clinical outcomes in idiopathic Parkinson’s disease adjusted for age at assessment and disease duration. Forrest plot with estimated coefficients and corresponding confidence intervals (±1.96 x Standard error) for sex, from linear/logistic regression of numerical/binary outcome on disease duration, AAA, and sex (binary outcomes are annotated by asterisk). The color blue indicates significant negative effects of male vs. female sex on the clinical outcome, and the color red indicates significant positive effects at the Bonferroni-adjusted 5% level. Clinical symptoms and scales are described in the Supplementary Material.

Clinical and demographic characteristics and outcomes of sex-stratified iPD are shown in Table 3. We did not observe a significant effect of male sex on the frequency of pRBD in iPD. Interestingly, from all the putative variables, only olfactory performance (measured by Sniffin’ Stick test) was significantly negatively, and FOG significantly positively associated with male sex in PD after adjustment for AAA and disease duration (see Fig. 4).

**Table 3:** Descriptive statistics for sex stratified iPD. Results are shown as mean and standard deviation (SD) for numerical variables, number of zeros (’NO’) and ones (’YES’) for binary variables and percentage of YES, and number of missing values (NA). The last column shows *p*-values from Mann-Whitney *U* test for numerical variables and Fisher’s exact test for binary variables. Binary variables are annotated by asterisk. Single and double ticks indicate significance at the 5% level and the Bonferroni-adjusted 5% level. Age at onset was calculated based on the year of the PD diagnosis

	PD female (*n* = 130)			PD male (*n* = 270)		
	Mean or	SD or	NA	Mean or	SD or	NA	*p*
	YES in %	NO/YES		YES in %	NO/YES
Disease duration since diagnosis (y)	5.44	5.53	0	5.35	5.46	0	8.1e-01
Age at assessment (y)	66.71	10.74	0	66.95	10.97	0	9.1e-01
Age at onset (y)	61.30	11.03	0	61.62	12.11	0	8.6e-01
H&Y	2.21	0.84	1	2.20	0.75	1	9.3e-01
MDS-UPDRS III	33.49	18.03	2	34.17	15.81	5	4.3e-01
MDS-UPDRS II	11.09	8.38	2	11.40	8.04	4	4.7e-01
LEDD (g/day)	0.47	0.36	0	0.55	0.42	0	1.2e-01
Gait disorder*	50%	65/65	0	58%	113/157	0	1.3e-01
Repetitive falls*	20%	104/26	0	16%	227/43	0	3.2e-01
MDS-UPDRS IV	1.90	3.61	4	1.77	3.30	1	9.3e-01
Dyskinesia/day (h)	0.87	3.22	1	0.63	2.55	0	8.6e-01
OFF time/day (h)	0.55	1.91	2	0.48	1.10	0	7.3e-01
Dystonia/day (h)	0.035	0.17	2	0.052	0.24	0	1.0e-01
Dyskinesia*	12%	115/15	0	13%	234/36	0	7.5e-01
Motor fluctuations*	11%	116/14	0	19%	219/51	0	4.3e-02^′^
Freezing of gait*	13%	113/17	0	26%	199/71	0	2.9e-03^′^
MoCA	24.92	3.84	3	24.41	4.24	4	3.4e-01
Sniffin’ stick test	9.10	3.26	4	7.76	3.30	6	2.2e-04^′′^
PDQ-39	43.28	26.38	8	37.92	26.26	10	4.0e-02^′^
SCOPA-AUT	14.92	8.01	2	14.83	8.08	0	1.0e+00
MDS-UPDRS I	10.22	6.32	3	10.14	6.96	7	5.5e-01
BDI-I	11.20	7.75	4	9.47	6.71	6	2.9e-02^′^
Starkstein Apathy Scale	13.84	5.77	6	13.86	5.60	1	9.7e-01
PDSS	102.64	25.08	4	106.68	22.94	3	1.3e-01
Probable RBD*	26%	96/34	0	35%	175/95	0	8.6e-02
Excessive daily sleepiness*	20%	104/26	0	33%	180/90	0	6.7e-03^′^
Insomnia*	27%	95/35	0	21%	212/58	0	2.6e-01
Hallucinations*	16%	109/21	0	15%	229/41	0	8.8e-01
Impulse Control Disorder*	7%	121/9	0	10%	242/28	0	3.6e-01
Orthostatic hypotension*	27%	95/35	0	27%	197/73	0	1.0e+00
Dysphagia*	26%	96/34	0	23%	209/61	0	4.5e-01
Constipation*	40%	78/52	0	42%	157/113	0	7.5e-01
Urinary Incontinence*	30%	91/39	0	31%	185/85	0	8.2e-01

### Effect of education and number of spoken languages on cognitive performance

We analyzed a potential confounding effect of the years of education (YoE) and the total languages spoken (TLS) on cognitive performance in our dataset. As shown in the Supplementary Table 1, only YoE (not TLS) had a significant positive effect on Montreal Cognitive Assessment (MoCA) in a multiple regression model adjusted for AAA and disease duration.

## DISCUSSION

The results of our study support the classification of RBD as a distinctive characteristic of the body-first subtype by identifying a significant association of iPD pRBD with the non-motor dominant disease profile, a result that matched remarkably well with the majority of previous studies [4–8]. It favors the concept of pathological process beginning in the peripheral nervous system with further centripetal spreading of alpha-synuclein in a subgroup of PD patients and hence the associated neurodegeneration causing a significantly higher autonomic dysfunction, higher depression burden as well as hallucinations through dysregulation of dopaminergic and noradrenergic system in the brainstem. Although we assessed RBD via a screening questionnaire, our results were consistent with a prior study using PSG-proven RBD, which indicated an association of a non-motor dominant phenotype in PD with PSG-proven RBD [4]. However, we observed only a trend in the negative effect of pRBD on global cognitive performance in PD, which did not correspond to several cross-sectional and longitudinal studies [8]. To assess a potential independent variable influencing cognitive performance, we identified a protective effect of YoE on cognitive decline in the overall PD group, but we did not identify a significant difference in pRBD PD vs. non-pRBD PD in terms of YoE or TLS. Therefore, we did not consider these two factors (YoE and TLS) as confounding factors for the effect of pRBD on cognitive performance assessed by MoCA in our dataset. Moreover, the *APOE* ɛ4 genotype, known to exacerbate beta amyloid pathology in Alzheimer’s disease, has been suggested to play a role in accelerated cognitive decline in PD [27, 28]. As RBD was associated with a higher rate of cognitive decline and dementia in previous studies, we explored a potential association between pRBD and *APOE* ɛ4 carrier status. However, no significant association between the two was observed in our study. This would argue for an independent effect of pRBD and *APOE* ɛ4 status without a synergistic effect on cognitive decline in iPD. Therefore, we conclude that *APOE* ɛ4 genotype might not play a role as a stratifier in body-first vs. brain-first concept. It is important to stress that we excluded a potential effect of PD-linked genetic mutations and genetic risk factors for PD, which may have contributed to confounding effects on clinical phenotype in other studies, as in the case of highly prevalent mutations in the GBA gene [29].

Our investigation of potential sex-related differences in iPD phenotype did not reveal a significant association between pRBD and male sex, as suggested by several prior studies using either a similar screening questionnaire approach or PSG [30–32]. This adds to the open debate about whether there are significant differences in the prevalence of RBD in males vs. females. We would like to point out that the higher frequency of RBD in males was observed in studies using the dataset of individuals referred primarily to sleep laboratories which may cause a referral bias, given the fact that males are reported to have more violent RBD symptoms and are therefore more likely to be referred for PSG [33–36].

Next, we studied the potential confounding effects of sex on other motor and non-motor symptoms. We observed a higher frequency of males vs. females in the overall PD group (67.5% vs. 32.5%), in line with the results from recently published large cohort studies [37–39]. Interestingly, we found only olfactory dysfunction and FOG to be positively associated with males, while other putative motor and non-motor outcomes showed no significant associations with sex after multiple testing correction. These findings might indicate that sex does not play a substantial role in defining the phenotype of iPD and thus do not account for the phenotypic differences associated with pRBD.

Our study displays several specific strengths: (i) a large dataset was analyzed relative to previous studies; (ii) PD cases were genetically stratified by NeuroChip and targeted sequencing of GBA, avoiding a potential confounding by PD-causing mutations that are known to significantly influence the clinical phenotype; (iii) the study design included all disease stages of PD regardless of the cognitive status, and (iv) a monocentric data collection assured the consistency of the dataset.

Conversely, some limitations of our study should also be noted: We investigated the research questions using a cross-sectional analysis, and further studies on longitudinal data are still warranted. Additionally, RBD was not assessed by gold standard PSG but by a more accessible method using a screening questionnaire, potentially including in part false positive patients for RBD with another sleep pathology. Furthermore, the presence of hallucinations might be wrongly considered by the patients to classify as RBD symptoms. Nevertheless, the association of RBD in PD with hallucinations has been widely reported in the literature [40–42], thus we do not consider the significant positive association of pRBD with hallucinations in our dataset as a potential mis-classifier of pRBD vs. non-pRBD. Finally, we did not have complementary data on the time relation between pRBD and PD, i.e., describing whether pRBD preceded PD or evolved during the clinical phase of PD.

However, the overall concordance of the results on the association of pRBD in PD with a non-motor dominant phenotype indicates that applying RBDSQ may provide a useful tool for patient stratification in future studies and clinical trials. It might prove to be a clinically relevant mean to screen for pRBD during the regular follow-up of PD patients in order to personalize and adapt the therapy and its potential secondary effects by the treating physicians. Finally, this study adds to the prior body of evidence that PD subtyping, in general, may serve the patient by providing treatment-relevant phenotype-genotype stratifications as a tool for future clinical trials.

## Supplementary Material

Supplementary Material

## DATA AND CODE AVAILABILITY STATEMENT

The dataset for this manuscript is not publicly available as it is linked to the Luxembourg Parkinson’s Study and its internal regulations. Any requests for accessing the dataset can be directed to request.ncer-pd@uni.lu. The code for the statistical models is available at: https://doi.org/10.17881/sw04-1w80.
