# Supplementary material for: Body-First Subtype of Parkinson’s Disease with Probable REM-Sleep Behavior Disorder Is Associated with Non-Motor Dominant Phenotype
Source: J Parkinsons Dis. 2022 Dec 16;12(8):2561–73. doi: 10.3233/JPD-223511 (PMC9837682; doi:10.3233/JPD-223511)
Supplement: Supplementary Material [file jpd-12-jpd223511-s001.pdf]

Supplementary Material

Body-First Subtype of Parkinson's Disease with Probable REM-Sleep Behavior Disorder Is Associated with Non-Motor Dominant Phenotype

Supplementary Table 1. Estimated coefficients from linear regression of Montreal Cognitive Assessment (MoCA) score, age at assessment (AAA), disease duration, years of education (YoE) and total languages spoken (TLS) in iPD.

	Intercept	AAA	Disease duration	YoE	TLS
PD model 1 (YoE)	31.22	-0.14′′	-0.12′′	-0.26′′	-
PD model 2 (TLS)	35.63	-0.16′′	-0.12′′	-	0.08
PD model 3 (YoE and TLS)	31.31	-0.14′′	-0.12′′	-0.26′′	-0.06

Model 1 investigates effect of YoE on MoCA adjusted for AAA and disease duration; model 2 investigates effect of TLS on MoCA adjusted for AAA and disease duration and model 3 investigates effect of both YoE and TLS on MoCA adjusted for AAA and disease duration. Single and double ticks indicate significance at the nominal 5% level and the Bonferroni-adjusted 5% level.

Clinical symptoms and scales

Movement Disorder Society-Unified Parkinson's disease Rating Scale (MDS-UPDRS I-IV) and Scales for Outcomes in Parkinson's Disease-Autonomic questionnaire (SCOPA-AUT) are used under the license number (14017_ND). Clinical outcomes dystonia/day, OFF time/day, were based on sub-items of MDS-UPDRS IV 4.6.2, 4.3.2. and 4.1.2. respectively. Assessment of sleep quality was done via Parkinson's Disease Sleep Scale (PDSS) [1]. The calculation of levodopa equivalent daily dose (LEDD, reported in g/day) was based on established conversion factors [2]. *De novo* patient is defined as a dopaminergic drug naïve patient not more than one year since diagnosis of PD. Definition of constipation corresponds to the diagnostic criteria ROME III and information was acquired in a semi-structured interview [3]. The Hoehn and Yahr scale (H&Y) corresponds to the modified version of the scale [4]. Quality of life was assessed via Parkinson's disease questionnaire 39 (PDQ-39) [5]. Depression symptoms were reflected by Beck Depression Inventory Version I (BDI-I) [6]. Olfactory function was examined with 16 items Sniffin' Stick test [7]. Cognitive performance was assessed via Montreal Cognitive Assessment (MoCA) [8]. Presence of recurrent orthostatic hypotension was assessed using a semi-structured interview inquiring about the symptoms of orthostatic hypotension, i.e., faintness, dizziness, light-headedness, vertigo, hearing disturbance, visual disturbance or syncope following the tilting,

standing up or after a long-standing relieved by sitting down or laying down. Symptoms included in the analysis were assessed during a semi-structured interview of the participant and/or the participant's proxy with a study physician and refer to the current motor and non-motor symptoms at the time of assessment. Urinary incontinence corresponded to any type of urinary incontinence, i.e., stress, urge, overflow or mixed urinary incontinence).

List of genes and pathogenic PD causing variants excluded from the dataset used for the analytical models

Heterozygote GBA p.N409S; heterozygote GBA p.E365K; homozygote GBA p.E365K; GBA p.T408M; heterozygote GBA p.L444P; heterozygotes GBA p.R398X; GBA p.G241R; heterozygote GBA p.A215D; heterozygote GBA c.115+1G>A; heterozygote LRRK2 p.G2019S; homozygote PINK1 p.L369P; heterozygote GBA p.H294Q; heterozygote LRRK2 p.G2019S; dual heterozygote of LRRK2 p.R1441C and GBA p.E365K.

REFERENCES

[1] Chaudhuri KR, Pal S, DiMarco A, Whately-Smith C, Bridgman K, Mathew R, Pezzela FR, Forbes A, Högl B, Trenkwalder C (2002) The Parkinson's disease sleep scale: a new instrument for assessing sleep and nocturnal disability in Parkinson's disease. *J Neurol Neurosurg Psychiatry* **73**, 629–635.

[2] Tomlinson CL, Stowe R, Patel S, Rick C, Gray R, Clarke CE (2010) Systematic review of levodopa dose equivalency reporting in Parkinson's disease. *Mov Disord* **25**, 2649–2653.

[3] Longstreth GF, Thompson WG, Chey WD, Houghton LA, Mearin F, Spiller RC (2006) Functional bowel disorders. *Gastroenterology* **130**, 1480–1491.

[4] Goetz CG, Poewe W, Rascol O, Sampaio C, Stebbins GT, Counsell C, Giladi N, Holloway RG, Moore CG, Wenning GK, Yahr MD, Seidl L (2004) Movement Disorder Society Task Force report on the Hoehn and Yahr staging scale: status and recommendations. *Mov Disord* **19**, 1020–1028.

[5] Peto V, Jenkinson C, Fitzpatrick R, Greenhall R (1995) The development and validation of a short measure of functioning and well being for individuals with Parkinson's disease. *Qual Life Res* **4**, 241–248.

[6] Beck AT, Ward CH, Mendelson, M, Mock J, Erbaugh J (1961) An inventory for measuring depression. *Arch Gen Psychiatry* **4**, 561–571.

[7] Hummel T, Kobal G, Gudziol H, Mackay-Sim A (2007) Normative data for the "Sniffin' Sticks" including tests of odor identification, odor discrimination, and olfactory thresholds: an upgrade based on a group of more than 3,000 subjects. *Eur Arch Otorhinolaryngol* **264**, 237–243.

[8] Nasreddine ZS, Phillips NA, Bédirian V, Charbonneau S, Whitehead V, Collin I, Cummings JL, Chertkow H (2005) The Montreal Cognitive Assessment, MoCA: a brief screening tool for mild cognitive impairment. *J Am Geriatr Soc* **53**, 695–699.
